# Pedagogical intentionality in walking football: fostering sustained participation and development among older adults

**DOI:** 10.3389/fspor.2026.1810949

**Published:** 2026-07-09

**Authors:** João Gustavo Batista, Júlia Barreira

**Affiliations:** School of Physical Education, University of Campinas, Campinas, Brazil

**Keywords:** active aging, older adults, pedagogical practices, soccer, sports

## Abstract

The growing population aging demands physical practices that promote health, inclusion, and autonomy. In this context, Walking Football (WF) emerges as an adapted sport for older adults, characterized by the absence of running and physical contact, creating a safe and inclusive environment. Although the physical, psychological, and social benefits of WF are well documented, less is known about how pedagogical intentionality is enacted in practice and how it supports sustained participation and development. Therefore, this study examined how pedagogical intentionality was enacted through pedagogical mediation and the construction of a learning environment in a WF program for older adults. This study was conducted with twelve participants aged 50 and older with no prior experience in the sport, who participated in a WF sports project for eight months, with sessions held twice a week. Data were collected through semi-structured interviews and analyzed using Reflexive Thematic Analysis. The findings indicate that sustained participation and development were supported by dialogic pedagogical mediation, continuous adaptation, and the intentional design of a supportive learning environment. Adapting activities proved essential to ensuring safety and equitable participation. Fundamentally, the promotion of a welcoming environment strengthened autonomy and confidence, while social bonds emerged as the central element for motivation. It is concluded that intentional and pedagogical mediation practices transform the practice, reinforcing the sport not merely as an adapted sport, but as a space for learning and social belonging for active aging.

## 1 Introduction

Population aging is a global phenomenon (1) that presents important public health challenges, including increased prevalence of chronic diseases, social isolation, and functional decline (2, 3). At the same time, high levels of physical inactivity continue to be observed among older adults (1). In this context, promoting health and active aging has become increasingly important, not only to extend life expectancy but also to support autonomy, functionality, and quality of life throughout later adulthood (3). As a response to these challenges, Walking Football (WF) has emerged as a promising strategy (4). Created in England in 2011 (2), WF is an adapted form of football specifically designed to facilitate the participation of older adults (5). Through modifications such as the prohibition of running, overhead play, and direct physical contact, the sport seeks to create a safe, accessible, and inclusive environment for participation (3).

Scientific literature has pointed to WF as a multidimensional intervention that combines aerobic activity, neuromuscular stimulation, and social engagement (4). Studies have shown significant benefits for physical health (cardiovascular fitness, strength, and fat reduction) (6–8), mental health (stress reduction), and social health (reduction of isolation and strengthening of social bonds) (2). However, although the benefits of participation are increasingly well documented, considerably less attention has been devoted to understanding how these experiences are pedagogically organized and facilitated in practice. More specifically, little is known about how pedagogical intentionality is translated into pedagogical mediation and the construction of learning environments that accommodate the diverse needs, experiences, and capabilities of older adults. Existing studies frequently emphasize the inclusive potential of WF and the importance of adaptation, yet they rarely examine how dialogue, participant agency, activity design, and the continuous adaptation of tasks, rules, and social interactions shape participants’ experiences within these programs. As a result, there remains limited understanding of how pedagogical mediation contributes to creating learning environments that support not only participation, but also sustained participation and development among older adults.

From a Sport Pedagogy perspective, sport can be understood as more than a context for physical activity; it can also function as a space for learning, autonomy, social connection, and personal development. Guided by this perspective, the present study draws on Game Pedagogy (Pedagogia do Jogo – PJ), a Brazilian pedagogical approach aligned with Game-Based Approaches (GBAs) (9), to examine how pedagogical intentionality is enacted within a WF program for older adults. Therefore, the aim of this study was to examine how pedagogical intentionality was enacted through pedagogical mediation and the intentional design of the learning environment in a Walking Football program for older adults, and how these processes contributed to sustained participation and development.

## 2 Pedagogical framework and principles of pedagogical mediation

The adoption of PJ is particularly relevant to the Brazilian context because it draws inspiration from street football as a significant cultural and educational practice (10). Rather than being governed by rigid structures, street football is characterized by self-organization, collective negotiation, and the continuous adjustment of spaces, rules, and materials according to the needs of those involved and the demands of the game itself (11). In such contexts, development occurs through interaction, experimentation, and participation, creating opportunities to improve perceptual, decisional, and tactical competencies (12). Equally important, its informal nature nurtures a sense of independence and emotional connection with the activity, factors that may contribute to long-term involvement in sport (12). These characteristics reinforce the value of creating sporting experiences that encourage exploration, negotiation, and adaptability. This rationale also explains the incorporation of Non-Linear Pedagogy (NLP), as both perspectives emphasize the influence of context and the purposeful manipulation of constraints in shaping meaningful experiences (13).

A central objective of PJ is the promotion of autonomy (10, 14). Here, autonomy is not interpreted as freedom from rules, but as the capacity to critically reflect, make decisions, and respond to the challenges that emerge during participation (15). From this perspective, the emphasis lies in progressively encouraging individuals to assume a more active role in shaping their own experiences rather than depending exclusively on external guidance. Consequently, PJ departs from the “banking concept of education” (9), embracing a horizontal and humanizing perspective inspired by Freirean thought (13). Knowledge is therefore constructed collectively through exchange, reflection, and dialogue (9). The educator's responsibility is not simply to transmit content, but to create opportunities that stimulate curiosity, critical thinking, and the search for multiple responses to the problems posed by the game (12, 15). Through this process, autonomy becomes evident not only in tactical decisions, but also in participants’ sense of agency, confidence, and ownership of the sporting experience.

The relevance of this framework to the present study lies in its understanding of pedagogy as a relational and dialogic process. Rather than prioritizing the transmission of knowledge, PJ emphasizes the creation of conditions that encourage inclusion, meaningful involvement, and shared responsibility (14). In practice, this requires continuously adjusting activities, rules, and interactions to accommodate participants’ needs while fostering a sense of belonging within the group. Such a perspective connects the emancipatory principles of Freirean pedagogy (9) with the goals of active aging, highlighting dialogue, social connection, and participant agency as essential elements for both personal development and continued involvement in sport (15).

Guided by these principles, the WF program was intentionally designed to translate theory into practice. Concepts such as autonomy, dialogue, participant agency, and game-centered experiences informed the organization of sessions, the role adopted by the educator, and the design of activities throughout the intervention. The program sought to recreate key characteristics of street football, including adaptability, negotiation, inclusion, and active involvement, within a structured context for older adults. The following section describes how these principles were operationalized through the organization of the program, its participants, and the pedagogical format adopted.

## 3 Learning environment, objectives, and pedagogical format

### 3.1 Learning environment and participants

This study was conducted at a university in the state of São Paulo, Brazil. The program was conceived as a learning environment designed to promote sustained participation and development through WF practice. Sessions took place in an indoor gymnasium, ensuring physical safety as well as access to restrooms and hydration facilities. The intervention was implemented across two cycles (March–June and August–November), totaling 32 weeks. Participants attended two 90-minute sessions per week.

The program initially involved 15 participants; however, 12 participated in the interviews due to availability during the data collection period. The participants consisted of four men and eight women aged 50 years and older. They presented diverse sporting and physical activity backgrounds. All four men had previous experience with football, although none had practiced regularly for several years. Among the women, two had played football during childhood, whereas six had no previous football experience. Importantly, none of the participants had prior experience with Walking Football.

Following approval by the University Ethics Committee (CAAE: 77402424.5.0000.5404), all participants received information about the study and provided written informed consent before participation. Data collection, processing, and storage followed General Data Protection Regulation (GDPR) guidelines, ensuring anonymity and confidentiality.

### 3.2 Pedagogical intervention and development objectives

Aligned with the theoretical principles described previously, the pedagogical intentionality underpinning the program extended beyond the promotion of physical activity and health. The intervention sought to create meaningful sporting experiences among older adults through engagement in WF. Drawing on the framework proposed by Côté and Gilbert (16), development was conceptualized through four interconnected dimensions that later informed the interpretation of participants’ experiences. Competence encompassed the development of game-related abilities, including spatial awareness, tactical understanding, positioning, and decision-making. Confidence referred to the development of self-efficacy, self-confidence, and the ability to overcome insecurities and anxieties associated with participation. Connection captured the development of meaningful interpersonal relationships, including friendship, mutual support, social interaction, and a sense of belonging. Finally, Character involved respect, empathy, cooperation, responsibility, and collective commitment to maintaining a safe and inclusive sporting environment. Together, these dimensions provided a framework for understanding how participants experienced development throughout the program and which factors contributed to their sustained participation.

### 3.3 Pedagogical format

The pedagogical format was intentionally designed to translate the principles of PJ into practice. Sessions followed a structure composed of three interconnected phases, creating a supportive learning environment while allowing flexibility for adaptation according to participants’ needs. The initial phase consisted of a warm-up that combined collective dynamic stretching with a 4v1 possession rondo. The complexity of this activity was progressively adapted through modifications in task constraints, such as changing the number of defenders or introducing cognitive challenges. One example was the “verbal rondo,” in which participants were required to associate passes with specific verbal categories (e.g., fruits or words beginning with the same letter), encouraging engagement and decision-making.

During the development phase, learning tasks were designed to promote participation through game-based situations. One example was the “zone game”, in which the court was divided into three lanes and players in possession were required to occupy all sectors of the playing area. Adaptations to rules, space, and player distribution created tactical challenges and encouraged participants to explore different solutions while interacting with teammates and opponents. Rather than emphasizing technical execution in isolation, the activities sought to promote learning through participation in meaningful game situations.

The final phase consisted of a 6v6 game without goalkeepers. To ensure safety, a restricted area was established near the goals to reduce crowding and minimize the risk of falls or physical contact. During this phase, pedagogical interventions were intentionally limited, allowing participants greater autonomy and preserving the flow of the game.

Across the intervention, learning experiences were organized progressively, moving from smaller to larger numbers of participants. This progression sought to support participants’ understanding of spatial occupation, collective organization, and decision-making within the game. Consistent with the principles of PJ and NLP, the intentional manipulation of task constraints constituted a central strategy for shaping learning experiences.

Pedagogical mediation throughout the program was characterized by active listening, dialogue, and continuous adaptation. Participants’ suggestions were considered throughout the intervention, and activities were modified when necessary to respond to emerging needs. Particular attention was given to explaining and revisiting rules patiently, respecting different learning rhythms and previous sporting experiences. Adaptation therefore functioned not as an occasional strategy but as a central pedagogical principle. For example, floor markings were used to organize space, support tactical understanding, and reduce anxiety during game participation. Similarly, rules and activities were continuously adjusted to ensure that participants with different physical capacities could engage meaningfully in the experience.

Finally, the pedagogical environment extended beyond formal session time. Social gatherings, including end-of-semester lunches, a bingo event, birthday celebrations, and a surprise Teacher's Day gathering organized by participants, formed an important part of the program experience. These moments strengthened interpersonal relationships, reinforced participants’ sense of belonging, and contributed to the development of a supportive social network. Consequently, the program became not only a space for sport participation but also a meaningful environment for social connection, mutual care, and active aging.

Although the educator had previous experience in football and futsal, this represented his first experience with WF. Consequently, pedagogical planning and decision-making involved an ongoing process of reflection, literature consultation, and adaptation. This reflective process was further supported through weekly meetings with the second author, which provided opportunities to discuss both the evolution of the intervention and the development of the research project.

### 3.4 Researcher positionality and reflexivity

In this study, the dual role of the researcher as both educator and primary investigator is acknowledged as a potential source of bias, particularly due to the close relationship with participants and the immersive nature of the intervention. This dual position creates an inherent asymmetry in the relationship between the educator-researcher and the participants, introducing the possibility of social desirability in participants’ responses. Given that the older adults were reflecting on a program led by someone closely involved in their weekly routine and with whom they had developed meaningful interpersonal relationships through both formal sessions and social gatherings, participants may have experienced some difficulty in openly criticizing aspects of the pedagogical mediation or the learning environment during the interviews. Recognizing that qualitative inquiry inevitably involves researcher subjectivity, explicit strategies were adopted to enhance the trustworthiness of the findings (17). Reflexivity was therefore treated as a continuous and integral component of the research process. The primary researcher engaged in ongoing critical reflection regarding pedagogical decisions, interactions with participants, and assumptions that could influence data interpretation, following recommendations for strengthening confirmability in qualitative research (18). To further reduce individual bias, regular meetings were held with the second author, creating opportunities for dialogic reflection, collaborative interpretation, and critical examination of emerging insights. Although formal member checking was not systematically implemented, participants were encouraged to openly express their perspectives throughout the interviews, and their experiences were represented through verbatim quotations to preserve the authenticity of their voices. Furthermore, the involvement of both authors throughout the analytical process contributed to investigator triangulation, strengthening the credibility and confirmability of the findings. Collectively, these procedures align with established criteria for qualitative rigor, particularly those emphasizing reflexivity, collaborative analysis, and the construction of trustworthy interpretations (19).

### 3.5 Evaluation and data analysis

This study adopts a qualitative and exploratory research design (20), to explore how pedagogical intentionality was enacted through pedagogical mediation within a WF program for older adults. The case was situated within a 32-week intervention and investigated as a contemporary phenomenon embedded in its real-world context. Rather than evaluating program effectiveness through predetermined outcomes, the study sought to develop an in-depth understanding of participants’ experiences and the meanings they attributed to their participation in the learning environment. Particular attention was given to how pedagogical mediation, the organization of learning experiences, and the broader social environment shaped participants’ perceptions of participation, learning, autonomy, and development throughout the program. Data collection occurred in person at two time points—after the fourth and eighth months of the intervention—through semi-structured interviews. The interview guide was organized into four thematic areas: presentation of the study, participant characteristics, experiences in WF, and perceptions of the pedagogical process. All interviews were audio-recorded and subsequently transcribed verbatim to support the data analysis. Given its flexibility and potential to deliver rich and complex understandings, Reflexive Thematic Analysis was chosen for data analysis (21). The analysis was conducted in six phases: i) familiarizing ourselves with the data; ii) generating initial codes; iii) searching for themes; iv) reviewing themes; v) defining and naming themes; and vi) producing the report (22). These steps were the product of a deep and prolonged data immersion and reflection, constituting an active and generative process (21). After conducting these six steps carefully, two central themes were identified from the interviews: “They listen to us; we talk and say what we like and what we don't like” – Dialogic Pedagogical Mediation in Walking Football: This theme explores the dialogic nature of the learning environment and how the session organization met the participants’ needs. “Here, I started smiling again” – Inclusion, Social Connection, and Autonomy Through Walking Football: This theme addresses the psychosocial impact of the practice and its role in fostering participant independence. Each theme was supported by verbatim excerpts from the participants and grounded in a solid theoretical framework.

## 4 Results and evaluation

### 4.1 *“They listen to US; we talk and say what we like and what we don't like*” - dialogic pedagogical mediation in walking football

The findings suggest that dialogic pedagogical mediation was experienced by participants as a defining characteristic of the learning environment, particularly in contrast to more directive and hierarchical sporting experiences. The opportunity to express opinions and be genuinely heard was not perceived merely as a pedagogical strategy, but as a condition that reshaped participants’ roles within the program. As expressed by WF-E, “They listen to us; we talk and say what we like and what we don't like,” indicating a shift from passive participation toward greater agency and involvement in the sporting experience. However, this participatory environment did not emerge without tension. Participants’ accounts indicate that the group initially operated within a more competitive and individualistic logic, “In the beginning, there was this competitiveness” (WF-R), suggesting that the construction of a collaborative environment required continuous pedagogical mediation. In this sense, dialogue functioned not only as a mechanism for inclusion, but also as a means of negotiating interpersonal relationships and collectively establishing values related to safety and mutual respect. This aspect was particularly relevant given participants’ age and concerns regarding physical integrity. As highlighted by WF-DJ: “The safety part, I think, was very interesting over time to maintain [our] physical integrity, since, at the very least, everyone is already close to 60.”

Participants also emphasized the importance of the structured organization of the sessions. However, the perceived value of this structure appeared to be linked less to the sequence of activities itself and more to the ways in which the learning environment was continuously adapted throughout the intervention. During the development phase, participants described how modifications in space and task constraints shaped their understanding of the game. As noted by WF-DJ: “One thing that is interesting is the dimensioning of the space on the court so you can move around.” The playing area was progressively expanded according to the group's development: “Throughout the semester [..] they increased the playing area as [..] they observed that people were more capable there” (WF-DJ). These adaptations suggest that participation and development emerged through the ongoing interaction between participants and the environment rather than solely through the acquisition of individual skills.

At the same time, these adaptations revealed differences in participants’ previous experiences, abilities, and learning rhythms. Activities such as the rondo (“bobinho”) were simultaneously perceived as accessible and challenging depending on the participant. As WF-DJA explained: “Today, for example, that ‘bobinho’ [rondo] we did. It's easy to do—well, for me it's easy, but for other people, it's difficult.” This highlights how a single learning task can generate diverse experiences within a heterogeneous group. To support participation and understanding, spatial markings were introduced as a pedagogical resource to organize movement and reduce crowding. According to the same participant, “Those squares we used on the court, right? To internalize that, to hold back a bit more, to give [ourselves] more time.” These adaptations helped participants regulate movement, positioning, and decision-making within the game. As illustrated by WF-E: “At first, everyone was on top of the person who had the ball. I learned to stay more spread out here, wait more for the ball, and be calmer.”

The prohibition of running emerged as a central tension within participants’ experiences. While it contributed to creating a safe environment, it also required significant behavioral adaptation. Participants described the need for “self-control” (WF-DJA) and a “struggle against anxiety” (WF-R), given the instinctive impulse to run: “[You] want to start running” (WF-DJ). These accounts suggest that adherence to the rules was not immediate, but involved an ongoing negotiation between previous bodily habits and the demands of the adapted game. In this context, pedagogical mediation played a crucial role, not by eliminating difficulty, but by creating conditions in which participants could gradually understand, accept, and manage these challenges as part of their learning experience.

Collectively, the findings indicate that dialogic pedagogical mediation, continuous adaptation, and the intentional design of the learning environment contributed to fostering confidence, participation, autonomy, and well-being. As expressed by WF-E, the experience “[It] soothes the emotional side,” while WF-S reflected: “Before, I felt insecure about playing ball; today I go, I move more, there are activities that help with balance and breathing”. Participants’ positive evaluations therefore extend beyond the activities themselves and point toward the importance of the learning environment that was intentionally constructed throughout the program. Rather than representing a straightforward validation of the program, the findings point to a more complex pedagogical dynamic in which dialogue, structure, and constraint manipulation interact in shaping participants’ experiences.

### 4.2 *“Here, I started smiling again”* - inclusion, social connection, and autonomy through walking football

Participants consistently described the learning environment as “very inclusive” (WF-DJ); However, this inclusivity appeared to be socially and relationally constructed rather than an inherent characteristic of WF itself. Factors such as age similarity among participants—“Everyone more or less in the same age group” (WF-V)—and a shared ethic of respect between individuals with different levels of sporting experience—“Those who have a bit more skill respect the people who have less” (WF-DJ)—contributed to reducing comparison, judgment, and exclusion. At the same time, participants’ accounts suggest that inclusion was not automatic, but emerged through the ongoing negotiation of relationships, expectations, and group norms throughout the program.

Within this environment, autonomy emerged as a central dimension of participants’ experiences. Importantly, autonomy extended beyond game-related decision-making and was expressed through opportunities for dialogue, freedom of expression, and active participation in the collective construction of the experience. As noted by WF-W, “Usually in sports the coach doesn't let you open your mouth; here, you can open your mouth,” highlighting a perceived contrast with more hierarchical sporting environments. Participants therefore experienced autonomy not only as the capacity to make decisions within the game, but also as a sense of agency and shared responsibility within the group.

Participants’ narratives further emphasized the importance of social interaction and affective exchange, positioning WF as a space of care and belonging. Expressions such as “It's not just football; it's the affection, the hug, the ‘good morning’ we give each other” (WF-V) suggest that the meaning of participation extended beyond the activity itself. For some participants, this relational dimension appeared to outweigh the sporting component, such as “I come more to see the people than for the exercise itself” (WF-ML), indicating that sustained participation was strongly associated with the social environment created throughout the program. In this context, social connection and psychological well-being appeared to be more meaningful motivators for continued participation than the physical benefits traditionally associated with sport and exercise. Participants’ accounts further reinforce the role of interpersonal relationships as a central driver of engagement. As WF-M stated, “I think the difference here, besides the sports practice, is being able to see the people who practice with us; it's a great motivator.” Through these experiences, physical activity was resignified from a potentially solitary obligation into a meaningful and enjoyable social experience. This transformation is illustrated by WF-DJA, who contrasted the experience with traditional exercise: “Walking is boring, isn't it?” while later noting, “Here we do aerobic activity while having fun.”

## 5 Discussion

The findings suggest that dialogic pedagogical mediation played a central role in shaping participants’ experiences within the WF program. Rather than functioning merely as a strategy for organizing activities, pedagogical mediation influenced how participants perceived their roles within the learning environment. Consistent with previous research, the quality of pedagogical relationships appears to be an important factor in sustaining participation and development among older adults (5). The adoption of dialogic practices aligns with both Freirean pedagogy and PJ by challenging hierarchical approaches and positioning participants as active contributors to the construction of the sporting experience (9). Within this environment, autonomy emerged not only through decision-making during game play but also through opportunities for dialogue and shared responsibility. However, the implementation of dialogic pedagogical mediation was not free from tensions. One challenge involved the redistribution of power within the learning environment. Facilitating participant voice required the educator to continuously negotiate authority and create opportunities for shared decision-making, a challenge also identified in previous studies adopting participatory pedagogies in sport (23, 24). A second challenge involved participants’ previous experiences with more directive and structured forms of sport participation. For some participants, adapting to a more dialogic and participatory environment initially generated uncertainty regarding expectations, responsibilities, and acceptable forms of involvement. These findings reinforce the idea that participant agency is not automatically produced by dialogic pedagogies but is gradually constructed through ongoing interaction and trust-building.

The heterogeneity of the group represented an important dimension of the pedagogical environment. Differences in gender and previous sporting experiences were particularly evident. While all men had previous football experience, most women had never practiced football before participating in the program. On one hand, this finding highlights the potential of mixed-gender WF environments to challenge historically gendered patterns of sport participation (25). It also contrasts with broader trends showing lower levels of physical activity participation among older women compared with older men (26). On the other hand, differences in sporting experience generated disparities in confidence, tactical understanding, and adaptation to the rules of WF. These differences were reflected in initial feelings of insecurity and in the competitive dynamics observed during the early stages of the program. In this context, pedagogical mediation played a crucial role in preventing exclusionary dynamics and supporting meaningful participation among individuals with diverse backgrounds (10). Through continuous adaptation, dialogue, and the collective construction of expectations regarding safety and respect, the learning environment became a space in which differences in experience were acknowledged without becoming barriers to participation. These findings reinforce the importance of viewing inclusion not as an inherent characteristic of WF, but as an outcome of intentional pedagogical action.

The adaptation of activities, rules, and learning tasks emerged as another central feature of pedagogical mediation. Consistent with the principles of PJ, adaptation was not understood as simplification, but rather as a means of recognizing participants’ diverse needs, experiences, and capacities (14). The prohibition of running illustrates this dynamic particularly well. Although participants recognized its contribution to safety, many also described anxiety, frustration, and difficulty adapting to this rule, findings consistent with previous research on WF participation (27). Rather than eliminating these challenges, pedagogical mediation focused on creating conditions that allowed participants to gradually understand and negotiate them. The use of spatial markings and modified game formats provides a practical example of this process. Through the intentional manipulation of task constraints, the educator shaped the learning environment in ways that supported understanding, reduced anxiety, and encouraged participation. Such strategies are consistent with both PJ and NLP, which emphasize the role of environmental design in supporting learning and development (12, 13).

A second major finding concerns the relationship between social connection, well-being, and sustained participation. Participants consistently emphasized the importance of interpersonal relationships and opportunities for social interaction. These findings align with previous studies identifying social connection as one of the strongest motivations for continued participation in WF (2, 4, 5). For many participants, the value of the program extended beyond the sport itself and was strongly associated with the social environment that emerged through regular interaction. Importantly, the findings suggest that well-being should not be interpreted solely as a consequence of participation in WF. Rather, well-being appears to emerge through the quality of relationships and experiences developed within the learning environment. Feelings of confidence, belonging, emotional safety, and renewed self-esteem were progressively constructed through repeated participation, supportive interactions, and opportunities for meaningful engagement. This interpretation is consistent with previous research highlighting the role of social support, belonging, and positive interpersonal relationships in promoting healthy aging (28).

Practical Implications and Lessons Learned: The findings suggest that the contribution of WF programs for older adults extends beyond the provision of an adapted sporting activity. Rather, their potential appears to reside in the pedagogical intentionality through which participation is organized and experienced. In this study, dialogic pedagogical mediation, active listening, and the intentional design of the learning environment emerged as central elements in supporting participants’ engagement, autonomy, sustained participation, and development. The findings highlight the importance of providing a structured session while maintaining sufficient flexibility to adapt activities according to participants’ needs, experiences, and capabilities. Within this process, the adaptation of rules, spaces, materials, and task constraints should not be understood as simplification, but rather as a pedagogical strategy for promoting safety, inclusion, confidence, and meaningful participation. Consistent with the principles of PJ and NLP, these adaptations can help create learning environments in which participants feel capable of engaging with challenges while maintaining enjoyment. The study also reinforces the importance of attending to the relational dimensions of participation. Social connection, opportunities for dialogue, and the cultivation of a welcoming and respectful atmosphere emerged as central factors supporting motivation, sustained participation, and development. Ultimately, this program is not universally effective; its implementation is inherently contextual and requires continuous pedagogical flexibility, defining WF not merely as an adapted sport, but as a complex educational space.

## Data Availability

The datasets presented in this article are not readily available because they are subject to specific ethical and institutional restrictions to ensure participant protection and research integrity. This study was conducted under the formal approval of the University Ethics Committee, and participation was strictly limited to individuals who provided written informed consent after being fully briefed on the study’s nature. To maintain the privacy and confidentiality of the twelve participants—comprising four men and eight women—all qualitative data collected through semi-structured interviews was transcribed verbatim and anonymized using alphanumeric codes such as WF-E and WF-DJ. These records were analyzed using Reflective Thematic Analysis within the institutional context of the University of Campinas (UNICAMP) outreach programs, specifically the School of Physical Education and the Universidade program. Consequently, the raw data is restricted to the specific academic purposes approved by the ethics committee and originally consented to by the participants to prevent any breach of confidentiality. Requests to access the datasets should be directed to JG, joaogu.batista@gmail.com.
